# Constantinople

**Published:** 1858-10

**Authors:** R. F. Foote

**Affiliations:** Member of the Imperial Medical Society of Constantinople.


### SANITARY AND SOCIAL SCIENCE.

### REPORTS OF CITIES, TOWNS, AND DISTRICTS.

### CONSTANTINOPLE.

By R. F. FOOTE, M.D., Member of the Imperial Medical Society of Constantinople.

WHEN as residents we visit the various parts of this great city, Constantinople, standing as it does a link between civilisation and barbarism; where the Divine nature has done so much and the human nature so little; two questions of importance present themselves to us as physicians; viz., the condition of its population in regard to health; and the peculiar effects of disease upon the population. We will consider, therefore, *seriatim*,

1. The climate and diseases of Constantinople, with the hygienic arrangements of the city.
2. The predisposing causes of disease and the amount of mortality.
3. The diseases prevailing and the means to prevent them.

The town of Constantinople, placed upon seven hills at the entrance of the Bosphorus, in very close proximity to the Marmora, and not twenty miles distant from the Black Sea, has, in and around it, a good climate, a picturesque neighbourhood, a light soil, a fine harbour, and a population from every part of the habitable globe,—speaking all languages, from Russian to Hindoostanee, from Italian to Circassian,—while wood and water, hill and lake, may all be said to be happily blended in and near this much coveted city. Situate in this position, the wind constantly blows from north to south, or in the contrary direction; all offensive material rapidly passes off in one course or the other.

There are no large towns above on the banks of the Golden Horn, whereby the collection of sewerage is sent down to infect the air, as we find in some towns of Europe. There are no large factories in its neighbourhood, engendering the worst forms of disease, not only physically on its population, but morally by their associations; no stagnant salt marshes are in its neighbourhood to taint the water.

The town of Constantinople has hitherto, however, been considered an unhealthy city; and, on examination of its streets, alleys, houses, means for supplying water, drainage, and cemeteries, this view will be but little altered. During the months of December, January, February, and March, many of its principal streets are impassable; thus, if we take the line of road from the English or Russian embassies to the Sublime Porte, a distance of perhaps two miles, we shall find that the streets will not admit of passage for waggons, carts, carriages, or other mechanical contrivances which the *genus homo*, as a rule in England, France, and America applies, to assist him in transporting the products of his own country to the sea for exportation, and to import those of other lands for his own use.

A combination of the level, inclined plane, lever, wheel and axle, are agencies which appear, as a rule, to be disregarded by the inhabitants of Constantinople.

To the Mussulman, as the ruler of the country, we must alone look as the person with whom to make arrangements, who will direct and enable others to put their powerful agents in motion. He is the person to form roads, the great arteries of the country, and keep those roads in a proper state of efficiency.

In examining the houses, we find that all the sanitary rules, generally considered of the utmost importance, are disregarded; that the dwellings are badly constructed as protectors against the cold and rain of winter, and are equally inefficient against the heat of the sun in summer; outside each door there is generally a heap of refuse, animal and vegetable, to be removed by the dogs, the only scavengers; or by the rains, which not frequently occur. The supply of water is very defective and insufficient, particularly during the summer, in Pera and Galata.

The mode of heating houses is by open charcoal fires, or, as they are termed, "mongals"; not an uncommon cause of death, I believe, particularly in Stamboul, where there are no means of producing heat by any other method.

The apartments are over crowded; and among the Christian population, several members of a family live and sleep in one room; numerous families inhabit one house, and at night they lie down in their day clothing.

There is no regular system of drainage; large cesspools are frequently observed near the houses, and the water-closet, or privy, is generally placed on the ground-floor beneath the living room.

With all these deficiencies, we are bound to consider Constantinople an average healthy city, a result due to a greater extent from its climate, than to any of the hygienic precautions practised by its inhabitants.

The same state exists at the various towns on the Black Sea; and where the natural conditions are imperfect, considerable amount of disease is always present; thus at Batoum and Sampson, there is always some form of fever.

However, in the midst of this city, gardens filled with luxuriant trees may be everywhere seen.

The vast amount of charcoal constantly consumed in the capital tends to assist more rapid decomposition, and to deprive the air of the various noxious principles; for the atmosphere has always an elasticity, a purity, and an exhilarating influence, which we have not witnessed in any other country. The absence of watery vapour in the atmosphere is proverbial in many parts of Pera, Stamboul, and the villages of the Bosphorus; this purity, we believe, is due to the amount of (ozone) electrical influence constantly present. Whatever may be the natural advantages, the artificial arrangements for health are extremely bad. The charcoal and ozone prevent the existence in a graver form of the various contagious diseases.

Although the baths in some parts of the city exhibit a style of architecture only equalled by the mosques and other public buildings, and their internal regulations and economy are very good, affording cleanliness at any time to the poorest inhabitants at a very low rate, yet we are inclined to believe, that the artificial heat acting on the epidermis tends much to cause the shrivelled countenance, and the premature appearance of old age, which are seen in the Eastern as compared with the Western peoples.

The value of baths as hygienic agents must be rather compared with the long and continued fasts of the Greeks and Armenians, and the daily abstinence during Ramazan of the Mussulman, together with the indulgence commonly practised. At other times, the inhabitants seldom change their body linen, and have a great horror of cold water, except to their faces, hands, and feet, as a daily custom.

Bleeding is performed here on each individual, with the same punctuality as bathing; and, no doubt, the nervous tone and energy is materially lowered by the custom of the inhabitants to submit themselves three or four times in each year to lose perhaps sixteen or twenty ounces of blood.

To the absence of a large quantity of nitrogenised food, to the use of olive oil as a regular article of diet, to the avoidance of exposure after dark to the falling dew, to the care of clothing themselves warmly, may be due the absence of many diseases. Pulmonary consumption, vesical calculi, and delirium tremens, are rare diseases among the population.

The practice of drinking strong decoction of coffee before going out in the morning, may be considered as a proper precaution against intermittent fever.

For a long time it has been found difficult to obtain any statistics as to the amount of disease, from the existence of so many communities represented by the different forms of religion, over which the chiefs watch with jealousy. The endeavour to procure any information is always difficult. In the absence of any published statistics by the Ottoman government, we take those by Mr. Kquesnel, who, in his *Essay on the Movement of the Population of Constantinople*, states—after making the calculation from the consumption of bread, meat, and other *parts*, collected by M. Verrolot—that the population of Constantinople, in 1848, consisted of 715,300; comprising the residents, the military, marines, the population passing between Constantinople, the suburbs, the Islands of Princes, and the chateau of the Seven Towers.

The resident families in 1848, figure as 557,000 souls; the Mussulmans figure as 340,000, or more than three-fifths of the whole population; the Rayas as 217,000.

The families thus contain 270,000 individuals of the male sex, and 286,160 of the female; this gives a return of 100 women for 94·6 men.

Among the Jews, the number of females surpasses that of the males by one-seventh. A preponderance is observed, but in a less proportion, among the Greeks and Catholics.

The Mussulmans and Armenians are those only among whom a larger number of males is represented than of females, viz., as 107 to 100; but the proportion is different when we include the slaves. We find 162,500 males for 177,500 females, which gives a percentage of 100 females for 91·5 males.

M. Verrolot states that it is in June, July, and August there is the highest rate of mortality, and in September, October, and November, the lowest.

The greatest number of births take place in January and February, instead of in September, November, and March.

Whatever may be the numerous causes which tend to affect the public health, it is not the less true, that it is more flourishing at Constantinople than in any large towns of Europe.

We are led to believe that the actual number of deaths, is one in 41 for the entire population, civil and military; this is nearly half that which has been observed at Vienna, and three-fourths of the mean mortality of the towns of Naples, Leghorn, Palermo, and Barcelona. This low mortality, he says, I ascribe:

1. To the salubrity of the climate.

2. The spreading of the population over a vast surface.

3. The vigorous constitutions of its inhabitants.

4. The vast number of unmarried females constantly being imported.

5. A simple regimen accompanied by great sobriety.

6. The absence of the higher intellectual occupations.

7. The non-existence of the miserable class of persons exhausted by excessive work; a miserable race which affect all the grand centres of industry.

The cause predisposing to disease among Europeans recently arriving in this city, is that of preserving the same regimen to which they were accustomed when living in their own climate.

The daily use of the same amount of nitrogenised food and carbonised drinks, I look upon as the chief causes of maladies in new comers to this country.

Take, for instance, a marine who has been accustomed to laborious exercise on board ship, stopping suddenly in the harbour of Constantinople; he is not at all acquainted with the fact, that it is necessary for him to take less food than he has been accustomed to; if anything, he takes more, because he receives fresh meat and vegetables, from which, for a longer or a shorter period, he has been deprived; he eats as much or more than usual; he has an attack of diarrhœa, congestion of the liver, or fever. Take, on the other hand, an European traveller, arriving at one of the principal hotels, fatigued with his journey, annoyed at his passage from Tophané to Pera; he indulges in three glasses of sherry, on the oily meats of the table, and after a few days he suffers from lassitude, from pain in the head, with constipation; or, on the other hand, he may have a dangerous attack of diarrhœa, or even continued fever. He immediately fancies this is a very unhealthy climate; whereas the disease ought really to be placed to his own imprudence, or to the neglect of his medical advisers, in not informing him of the necessity for abstinence on his arrival.

I have been repeatedly called to patients who fancied that they were suffering from some very serious malady; when, on the administration of a mild mercurial at night, followed by some slight aperient in the morning, in two days all the urgent symptoms rapidly disappeared, and the patient has soon felt himself perfectly able to walk to St. Sophia, or the New Palace of the Sultan, and receive benefit from the exercise.

